# Experiences and needs of carers of Aboriginal children with a disability: a qualitative study

**DOI:** 10.1186/s12875-017-0668-3

**Published:** 2017-11-29

**Authors:** Michelle DiGiacomo, Anna Green, Patricia Delaney, John Delaney, Patrick Patradoon-Ho, Patricia Mary Davidson, Penelope Abbott

**Affiliations:** 1 University of Technology Sydney, Faculty of Health, PO Box 123, Broadway, Sydney, NSW 2007 Australia; 2 Blacktown and Mt Druitt Hospitals, Western Sydney Local Health District, Blacktown Road, Blacktown, NSW 2148 Australia; 3 Johns Hopkins University, School of Nursing, 525 N. Wolfe Street, Baltimore, MD 21205 USA; 4 Western Sydney University, Locked Bag 1797, Penrith, NSW 1797 Australia

**Keywords:** Caregivers, Childhood disability, Aboriginal and Torres Strait islander, Indigenous, Qualitative

## Abstract

**Background:**

Australian parents/carers of a person with a disability experience higher rates of depression, more financial stress, and are twice as likely to be in poor physical health than the general population. Aboriginal and Torres Strait Islander peoples experience worse health, social and economic outcomes than other Australians, and those with a disability face ‘double disadvantage’. This study aimed to better understand the experiences and needs of parents/carers/families of Aboriginal children with a disability.

**Methods:**

Semi-structured in-depth interviews were conducted with parents or primary carers of Aboriginal children aged zero-eight with disability. Interviews were analysed using thematic analysis.

**Results:**

Nineteen women (sixteen mothers and three grandmothers) were interviewed. More than half were lone carers (without a partner or spouse). Participants described their experiences, including challenges and facilitators, to providing and accessing care, impacts on their health and wellbeing, and associated economic and non-economic costs of caregiving. Financial strain and social isolation was particularly prominent for lone carers.

**Conclusions:**

Tailoring services to the needs of carers of Aboriginal children with a disability means supporting kinship caregiving, facilitating engagement with other Aboriginal families, and streamlining services and systems to mitigate costs. The experiences described by our participants depict an intersection of race, socio-economic status, gender, disability, and caregiving. Services and funding initiatives should incorporate such intersecting determinants in planning and delivery of holistic care.

## Background

Increasingly, family carers of people with a disability are subsuming greater responsibilities while caring for people at home [1, 2]. They are often required to fill in the gaps in fragmented service and support systems through their caregiving [2]. Consequences of caregiving responsibilities for parents/carers include adverse health and social outcomes related to high levels of stress experienced in dealing with challenges of caring and juggling the demands of daily life [3, 4]. Parents/carers of a person with a disability have been found to experience higher rates of depression, stress, including financial stress, and are twice as likely to be in poor physical health compared to the general population [2, 3, 5]. Family relationships can also be strained as high stress in carers and concern for other household members are prominent reasons for decisions to place children in out-of-home-care (OOHC) [1, 3, 6].

### Influence of specific contexts on the caregiving experience

While similar impacts of caring for a person with a disability have been reported across populations, contextual differences highlight the need to explore the experiences of parents/carers/families within priority populations, or those who experience health inequities, as they may face unique and additional challenges [7]. Experiences of stigma towards disability, different conceptualisations of disability, personal factors including resilience, cultural variations in support-seeking behaviours, and variations in access to available support services and resources can impact caring [4, 7, 8]. Obtaining a better understanding of the caring experience in priority populations, is particularly important in facilitating access to supports [9].

### The Aboriginal context of caregiving

Aboriginal and Torres Strait Islander peoples are one such priority population who experience worse health, social and economic outcomes than other Australians [2]. Government policies of dispossession and racism have contributed to Aboriginal people being one of the most disadvantaged socio-economic groups in Australia. Chronic illnesses are much more prevalent among the Aboriginal population and they impact at younger ages. They are more likely than other Australians to experience poverty [10, 11], unemployment [12], make less money, and live in rented homes and homes that are overcrowded (National Aboriginal and Torres Strait Islander Social Survey [12]. These disparities are also apparent in disability throughout the life course. Aboriginal children aged 0–14 years are more than twice as likely as non-Indigenous children to have a disability. High educational attainment and employment is less likely in Aboriginal and Torres Strait Islander people with disability compared to non-Indigenous people with disability [13].

These disparities likewise affect Aboriginal and Torres Strait Islander carers who are more likely to be an unpaid carer for a person with a disability than non-Indigenous Australians [14]. The impact of caring is more pronounced for Aboriginal and Torres Strait Islander carers and their communities due to the high levels of socioeconomic and health disadvantage [15]. Furthermore, conceptualisations of caregiving are different in Aboriginal families where family obligation is part of the culture. Connotations of family extend to all relatives who are nurtured within a community context, as in a kinship system, and it is appropriate for families and communities to look after one another [16]. For example, many Aboriginal carers rely on the extended family to give them a break from caring rather than approaching formal (paid) services for respite [15]. Kinship care is currently the most common form of out-of-home-care (OOHC) for Aboriginal children [17]; this has specific implications on the impact of caregiving for Aboriginal kinship carers of a child with a disability. Also, the term ‘carer’ may not resonate with Aboriginal carers who perceive it as reflecting formal care workers; thus, Aboriginal carers may not identify as carers despite significant care responsibilities.

In addition to this culture of shared caring within families and communities, Aboriginal carers may not access formalised external supports for other reasons. Historical mistrust of government institutions and a lack of culturally acceptable services and resources mean that parents/carers may only seek help when there is a crisis [18]. Lack of cultural awareness and sensitivity in mainstream service providers can result in misunderstandings and fractured trust and relationships. Aboriginal carers may also be unaware or have little knowledge of services and supports, their eligibility requirements, and how to access them. Low income and lack of transport are additional barriers to services for carers.

Despite the disparity and the significant role played by carers, little attention has focused on the needs of Aboriginal parents/carers of a person with a disability. It is necessary to facilitate timely access of children with a disability and their families to appropriate health services, social support services, and treatment because addressing problems early is crucial in reducing long-term negative impacts on health, education and employment outcomes [19–21].

### Aim

We conducted this study to better understand the experiences and needs of parents/carers/families of Aboriginal children with a disability. Understanding the experiences and impact of caring on Aboriginal parents/carers/families is critical in establishing a baseline to inform improvement of services.

## Methods

This study was informed by a socio-ecological framework situating carers’ experiences at the macro- (government), exo- (organizational), meso- (provider), and micro (family/child) system levels in recognition of the inter-dependence of these factors. The carer, child, and family are positioned at the centre of the framework. This micro-level experience is the focus of this paper. Findings related to navigating systems external to the family and additional methodological detail are reported elsewhere [22]. We used a qualitative approach using thematic analysis underpinned by phenomenology to facilitate understanding experiences of carers for Aboriginal children with a disability. [23].The sampling frame consisted of participants who were parents or primary carers (hereafter, carers) of Aboriginal children aged zero to eight years who attended a developmental clinic at an Aboriginal health service in a suburban area near a capital city in eastern Australia. The clinic, run by a team of visiting paediatricians, Aboriginal Health Workers, nurses, and midwives, catered to children with developmental problems aged from birth to sixteen years. Problems managed in this clinic are similar to those seen in other developmental clinics, although it catered specifically to Aboriginal and Torres Strait Islander children. Recruitment was facilitated by clinic staff who verbally informed potential participants about the study and supplied written information to interested parties upon presentation to the clinic for scheduled appointments or during home visits. Sampling was purposive in that recruitment targeted carers of children attending the clinic. We used an inclusive definition of disability to intercept experiences relating to mild, moderate and severe, physical, intellectual, or developmental conditions. Recruitment continued until no new issues emerged during interviews.

### Data collection

Between April 2013 and June 2015, in-depth semi-structured interviews were conducted on two occasions within twelve months with each participant to capture a range of experiences and perspectives of caring for a child with a disability. Initial interviews were conducted at the health facility in private clinic rooms, were audio recorded, and ranged in duration from 20 to 60 min. Participants were reimbursed for travel and child care expenses incurred as a result of participation. Interview topics were derived from a literature review [24] and the social determinants of health and social capital frameworks [25–27]. Within the interviews, we explored participants’ meanings and interpretations of their experiences with the child’s disability, the ‘patient/family journey’ enacted to access services and support, and participants’ perceptions of the effects of the child’s disability and ‘journey’ on the carer and family.

Follow-up interviews were conducted with participants, in-person or via telephone, to document activities related to the carer’s experience since the first interview twelve months prior. For example, some participants described attending ongoing medical appointments or changes in living arrangements since the first interview and how they made sense of these experiences. This second interview was also an opportunity to summarise thematic content of the first interview to seek confirmation of its validity with participants [28]. Any noted discrepancies were discussed, clarified, and resolved to the satisfaction of the participant.

### Research team

The research team was co-led by Aboriginal and non­Aboriginal team members, who frequently met to discuss research processes and debrief about ongoing data collection. The core research team comprised four female researchers supported by a male Aboriginal elder (JD) who provided cultural mentorship to the team. One member of the research team was an Aboriginal woman from the local community with experience in health service delivery, Aboriginal health, and childhood disability (PD). Both these team members worked in the Aboriginal health service in health promotion roles. One of the researchers (MD) who conducted interviews and analysis was a qualitative researcher based at a university and had qualifications in psychology. She had had an 8-year relationship at the study site wherein she worked with staff on health promotion and secondary prevention program research, but was unknown to participants. Another interviewer and analyst (PA) was a general practitioner (GP) employed at the service and was a university-based academic. While she had met some of the participants in the context of primary care consultations, she was not involved directly in the child’s healthcare, and ensured that participants were aware that her role in the research was separate to her role as a GP at the service.

### Analysis

Thematic analysis began with development of a contact summary sheet for each interview with key demographic information and emergent issues noted [29]. Thematic analysis was used because our aim was to understand carers’ experiences and perceptions, yet we focused more on patterned meaning across the data-set [23], rather than prioritising the idiographic focus, as in interpretive phenomenological analysis. One researcher (MD) undertook preliminary analysis wherein she read transcripts to familiarise herself with the data, added notes to each document, including analytic observations for individual transcripts and as well as the entire set, and developed a coding system with semantic and latent codes to elucidate categories. Data relevant to each code was collated individually for each transcript and at the end of the coding process. To facilitate rigor, a second researcher (BA) independently coded unmarked transcripts. The full research team met regularly to discuss code development throughout the data collection and preliminary analysis stages; differences in coding scheme were discussed until consensus was reached. The interview transcripts were then coded according to the developed scheme with consideration of emerging categories. Categories were then collapsed into themes. Emerging themes were discussed with the full research team before analysis was finalised. To further enhance trustworthiness, a stakeholder check was undertaken, whereby preliminary analysis of findings was presented to clinic staff, some of whom were also Aboriginal carers of a child with a disability, for their feedback. Feedback was incorporated into theme development.

### Ethical considerations

Ethical approval was granted by the Aboriginal Health and Medical Research Council (AH&MRC) (762/10) and University Human Research Ethics Committee (UTS HREC 2011-417R). The study adhered to key principles for research with Aboriginal and Torres Strait Islander peoples as espoused by the AH&MRC [30]. Participants were made aware that all identifiable information would remain confidential and that they could withdraw from the study at any time and that this would not affect any relationship they may have with the health service or researchers. Permission to audio record interviews was obtained from each participant. To preserve anonymity and confidentiality, names were replaced with pseudonyms and identifying information was removed following verbatim transcription of recordings.

## Results

Participants were sixteen mothers and three grandmothers, of whom 53% (*n* = 10) were lone carers (without a partner), and were taking care of 60 children at home, half of whom were identified as having a disability or developmental delay (Table 1). Types of disabilities or disorders represented in the care recipients were predominantly developmental disabilities including intellectual disability, autistic spectrum disorder, ADHD, language and communication disorders and hearing impairment, as well as associated conditions such as epilepsy and genetic disorders.

**Table 1:** Demographic details of carers (*N* = 19)

Characteristic	N (%)
Female	19 (100)
Sole carer	10 (52.6)
*Relationship to child:*	
Mother	16 (84.2)
Grandmother	3 (15.8)
# of children with a disability/developmental disorder they care for	30
Average age of child with a disability/developmental disorder	6.4 years (Range: 5 months – 13 years)
Other children in their care	30

Participants described their experiences in terms of caring for their child and family, challenges and facilitators of this caring, carer health and wellbeing, and associated financial expenses and non-economic costs. Our focus on costs of caring arose from the coding process. This led us to seek out a framework to assist in exploring and organising these economic and non-economic consequences of caring [31]. The data reflecting explicit content are thus presented in tabular form (Table 2) to provide specific examples of how participants experienced each consequence in the framework and are summarised narratively below. Following the discussion of economic and non-economic costs incurred by informal caregivers, factors that influence these costs are presented.

**Table 2:** Economic and non-economic costs incurred by informal caregivers

Economic Costs	Employment Consequences	Labour force exit/preclusion	Well I was working. But I have stopped…Yeah, the amount of time that I have off. It was my decision, it’s the school, a handful of weeks like every day. But she has at least one day off here or, like, five days off. She had had two weeks off, you know like. (Nadia)
			when we got the one that was 13 weeks, (my husband) gave up his job and stayed with him. Virtually raised the third one. The first two we stayed at work and tried to cope, and then with the bad behaviour and that it just got a bit too much. And yeah, so I only work two days a week now, and my husband doesn’t work, he had got a carer’s pension for the children. (Bree)
		Restricted work hours/absences	It is hard with the boys. I work three days a week at the moment, because both the boys do speech therapy every week, and it is hard, but such is life. We’ve always done it. (Barbara)
	Out of Pocket Expenses	Purchases for Care Recipient: supplies	money is a big issue for me too. Like, for me to have a house, raise my daughter and buy her medical needs, her clothing, her food, like, just anything in general, with the house, electricity and that, I’m pretty much looking at nothing at the end of when I get paid. Like, it’s not enough. I’m on a single parenting payment and I get the carer’s allowance. Not enough at all. (Samantha)
			…they ended up getting me an authority script for the [over-the-counter pharmaceutical] because it’s, like, $18 a box, but I was buying three boxes a week. And it’s got very expensive. Basically my husband works overtime to pay off our credit cards that pay for when I have been in hospital and you know we have to eat at the hospital, parking at the hospital, I am not entitled to anything. So, I don’t have a disabled sticker, I don’t have anything. (Nadia)
			Well, the problem is there is Centrelink won’t accept that she has a problem. I have been to them and [her condition] is not considered, so therefore I am eligible for nothing. So yeah, it’s quite frustrating…they tell me that they are looking into a healthcare card, but nothing else. She is not going to get – I am not going to get carers [support], I am not going to get support or anything like that because they don’t see it, they don’t recognise her problem as a problem. So that was the next thing Dr. X was sort of saying when we have the biopsy done maybe he can try again, but I try until I am blue in the face to tell them she has a problem, but it’s not on their list, so it’s not recognised. (Nadia)
		Purchases for Care Recipient: health services	So then I became a single mum, done this. Now I live in my own place where you’ve got to pay rent, it’s baby food, and – not baby food, nappies, food and all – I end up being broke. (Tabitha)
			Because you’re putting so much effort into getting them to different places, especially if you’ve got to pay privately as well, it really does hit the hip pocket. Um, sometimes it can make you go without your petrol because you’ve either got to weigh it up what is more important. (Rita)
			the therapy stuff was a lot – it’s a lot harder to access than – even with children that have very clear diagnoses and very clear needs, I mean unless you’ve got money to, sort of, get the private stuff. (Ainslee)
			I mean it’s – it’s significant because, especially, um, we did OT for a while through the kids’ hospital and the private one there, um, I mean the initial, the very first visit and it was literally half an hour, it cost – it was, like, $180… and you only got, like, $40 back. Like, it was really small… And then it was – I mean every subsequent half hour was, like, 50 bucks and you got $25 back, or something, so – but it’s that initial, sort of, um, cost - and plus travel and plus with young kids, you know…a lot of it was just play therapy and, you know, stuff that we do at home anyway, and I just thought, you know, it was such a waste of time and money. (Ainslee)
			he was seeing another paediatrician and I just could not afford to send him when he needed to go because it was like a hundred and something dollars. I know that you get money back but its $100 that you have to fork out to pay for it up front. (Grace)
			Cause it’s expensive. If they didn’t have a free service like this, what would they do?...You know, and living expenses – living. I – I get broke you know what I mean? But that’s with the circumstances of taking a pay cut and them sorts of things. (Laura)
		Transportation/travel	Costs me about a hundred and two dollars to fill up my car. And that means if I go to (hospital) more than four times a week I have to fill up again. (Lesley)
			There’s no way, I would never afford the petrol; it’s right out [distant suburb]. Then you’re stuck in traffic, you may as well wait out there at the gate [laughs], take a pillow. (Melanie)
	Caregiving labour	Time spent getting to services	You don’t pick the school they actually go to. Um, when they’ve got a certain level of disability where they need to be at a special school, um, you don’t get to pick…they decide where she fits best and where there’s space… Because there’s not very many specialist schools in Sydney…it’s an hour each way for us, basically…it makes it hard, and I think people just, sort of, take that stuff for granted that you just can’t send her to the school next door… you have to send her to the place that they think is going to give her what she needs…I mean it’s a really good school, and stuff. It was just the distance, and it’s not feasible for us to move closer ‘cause it’s more expensive to live close to there. (Ainslee)
			…when they have got appointments and they are all bang, bang, bang, and that’s a bit challenging because I have also got my own appointments and so I try and work around so it all fits in. (Stacey)
Non-economic costs	Physical Health and Wellbeing	Injuries/Physical Stress/Fatigue	they didn’t put [the child] on drugs, they put me on drugs … because she was so bad before I got here, um, I had a broken nose from her… I couldn’t touch her without her attacking me, yeah, really violent. (Jocelyn)
			It’s tiring and especially when I work nights… It’s tiring to, um, drag the kids around and – yeah…I mean it is - it’s very tiring, emotionally and physically. (Ainslee)
	Mental Health/Emotional Wellbeing	Depression, Anxiety/Psychological strain	And then, yeah, when she got diagnosed with it – it’s hard…I stress a lot, I cry a lot. (Rachel)
			It’s become very stressful, yeah. At the doctor’s today, before I was going to the hospital, I am on edge and I am, like, fighting doesn’t get it done, but I can’t just sit back and agree anymore and say, ‘Yeah don’t worry, you know it’s okay. We will see.’ (Nadia)
			I feel a little bit, you know, like last night, oh, my chest started getting a bit tight, you know…She takes out a lot of frustrations out on me and, well, I’ve got nobody to take mine out on, so I need my time out. I’ll lock myself out in the backyard if I have to. Lock myself in the toilet [laughs]. It’s only five minutes, but that five minutes is all you need to calm down. (Jocelyn)
			It is – it was very hard after I lost her dad. But I found it very – I’m finding it very hard to cope some days, and find it easy other days…[She was] shaking, and the eyes rolling back in her head, and all that. Yeah…I just wish that – it’s just like living in a nightmare. (Susan)
	Social Wellbeing	Activity engagement/Social Participation/Isolation	Yeah, I don’t take her anywhere anymore. I don’t take her food shopping. I don’t take her – like my aunt’s wedding was on the weekend… I don’t take her anywhere with me anymore because she’s just too full on and I can’t – it stresses me out at the shops when I’m trying to do shopping and I can’t – I can’t think what I need to get. (Rachel)
			I’ve moved down here [three years ago][to have better access to health services], I have been out bush for 20 years, so I’ve got no friends down here… (Jocelyn)
			I don’t have no support…I do everything on my own…Sit in the hospital all day by myself… I don’t get people coming to the hospital bringing me meals…I wish I just had a little bit more support, you know. Like, a little bit more. It’s depressing sitting in the house by yourself and having no-one to talk to. (Susan)
		Relationships	It’s affected it big time. Um, because I can’t go to see my grandkids anymore. Um, I worked, you know, and you have got to move around things looking after these two girls (Stacey)
			With my daughter, it was hard until I got her into a boarding school. She started Year 7 this year, and she’s over at [Sydney suburb]. So she just comes home on the holidays.(Barbara)
			My youngest daughter, she doesn’t live with me, she lives with my auntie because at the time I – like, I’m still a single mother but I can’t raise a child with a disability and a newborn on my own. It was way too much. My auntie, she can give, like, she can give my daughter a life I never could. (Samantha)
			You’re too ashamed to be able to tell family. Some think ‘oh, that’s just gammon, the ADHD...he’s just mucking up...just give them a hiding.’ (Laura)

### Costs incurred by caregivers

#### Economic costs

We organised findings reflecting economic and non-economic costs incurred by family caregivers using the taxonomies developed by Keating et al. [32] and Lero et al. [31](discussed below). Keating’s taxonomy depicts economic costs as employment consequences, out-of-pocket expenses, and caregiving labour, or time spent on caregiving and related activities. Excerpts reflecting each domain appear in Table 2 because they illustrate manifest content regarding caregiving costs. Caregivers referred to limiting or rearranging paid working hours or resigning from employment to accommodate their children’s needs. Inability to juggle demands of employment and attending multiple appointments, providing care on non-school days, and managing behaviour resulted in exiting the workforce. Out-of-pocket expenses involved purchasing supplies, accessing health services, and transport costs. Despite the context of universal health care coverage, accessing specialist or private health providers required a financial outlay considered significant in light of other household expenditures. Parking fees and meals were indirect costs of care-seeking. Financial pressure was unabated in carers receiving government subsidies. Conditions that were undiagnosed or not recognised as subsidiary-worthy translated to an increase in out-of-pocket costs for carers. Financial strain was particularly prominent for lone carers with only a single income. Related to both out-of-pocket expenses and caregiver burden were costs associated with traveling to and from multiple appointments and distant schools.

#### Non-economic costs

Lero’s three non-economic cost domains are physical, psychological, and social health and wellbeing [31]. Participants discussed the ways in which their physical and psychological health and wellbeing had been affected over the course of their caring role, from the period prior to diagnosis through ongoing management as the child aged. Stress reported by carers often centred around their child’s behavioural problems. Ongoing aggressive behaviour, even by very young children, challenged caregiving and caused injury to carers and siblings. The impact of concurrent stressful life events, such as the loss of a partner, further amplified the emotional impact of caring. In addition to sleep deprivation, worry, frustration, anger, and grief were described by participants. Carers’ other roles and relationships suffered as did self-management of their own health care needs. Family relationships were affected by the strains of caregiving and needs of the child with a disability in the family home. There were several examples of the physical separation, whereby the child with a disability or their sibling(s) were sent to live with other family members or to boarding schools to facilitate educational and social growth.

Lone carers, or those without a partner or spouse, were especially vulnerable to social isolation. The lack of emotional support was prominent in their narratives and it impacted on carers’ ability to cope with strain. Relocating to be closer to service networks sometimes hindered beneficial social support networks. Yet, feelings of isolation despite proximity of family sometimes surfaced due to lack of acknowledgement and understanding of the carers’ experiences.

### Factors that influence the impact of costs of caregiving

Lero et al.’s [31] framework for caregiving suggests that characteristics of the caregiver, care recipient, the relationship of the dyad, and the context and nature of care influence costs of caregiving and thereby challenge or facilitate resilience. Findings depicting the context and nature of care reflected latent content as they reported concepts underpinning the data.

#### Balancing needs of differently-abled children

Diverse needs related to learning, psychosocial support and development, healthcare, and safety all need to be balanced by carers of children with and without disability. Siblings and the family unit sometimes bore the brunt of the accommodations required. Concerns ranged from developmental, behavioural, and learning-related to the physical safety of siblings.

Younger siblings of a child with a disability occasionally modelled behaviours of the older brother or sister. For example, Ainslee was challenged by regressed speech and toileting behaviours in her son who does not have a disability or delay, unlike his older sister:

*“Certain things he’s very regressed in because he’s modelling himself from her and because she, sort of - toilet training’s a big issue for her - so it’s a big issue for him…And speech is an issue for him because he’s, sort of, learning speech from her … we try really hard not to treat our kids any different. I know there’s nothing wrong with him, he’s far more ahead than Sally was, but I worry that he’s going to learn bad habits and he’s going to not be able to stop.” (Ainslee)*.

Managing multiple children’s diverse needs could tax a carer’s resources, particularly a lone carer, and lead to choices about who missed medical care or school:

*“For the first two months of the baby’s life, it was a bit hard…So there were two months there where my kids didn’t get the services they needed.” (Barbara)*

*“Even if one of the kids has to go to the doctor, I have to get them all out of school.” (Helen)*

In several other cases, parents referred to their children without disabilities as missing out on attention:

*“…the other ones feel it. So you’ve got to make sure you make that time for them as well…And say, ‘No, no. We’re talking. Your time is later.’…But yeah. It’s so much attention around him.” (Laura)*

Some participants reported aggression perpetuated by their child with a disability either towards the carer or siblings. These violent interactions were described as frightening, disrupting family harmony, and shifting household dynamics:

*“He is now starting to slap [his sister] across her face, pulling her hair, and I’m trying to explain to him that is naughty. So now she’s got a big bruise on her face where he’s bit her.” (Tabitha)*

Physical aggression between siblings could jeopardize safety of family members and consequences reportedly included physical separation via changed living circumstances in some situations.

#### Family resources support the disability journey

Although caring for child with a disability impacts on the family, family also impacts on the caregiving and disability journey. The resources within families were used to provide respite, access services, afford instrumental and emotional support, and facilitate empowerment of carers.

For many families, respite is a family matter and using respite service rather than a family member or friend was perceived as unacceptable. They would not feel confident that an unknown respite provider would understand their child’s needs:

*“She can’t get minded by a lot of people because they don’t know what to do with her.” (Lesley)*

Many carers were fortunate to have family members willing to act in an informal respite capacity. However, planning for informal respite was difficult given multiple competing demands and at times, several children who need looking after.

*“We had family to help to look after the other ones while we have to do things for her, but, that’s still difficult depending on what times of the day and I work nights, so it definitely has a big impact.” (Ainslee)*

Even brief periods of care provided by trusted others afford the carer time to rest and restore.

*“I have a lot of support from my mother-in-law, like she’s really good… ‘cause I don’t get a break really from her and the only time I do is when my mother-in-law does take her for the night or something like that. She plays up for her, but my partner’s mum is very strict and won’t put up with it.” (Rachel)*

*“My sister goes, ‘Go outside, get a bit of fresh air, and come in when you are ready.’” (Tabitha)*

Examples of kinship care arrangements helping to keep separated families together were described. Despite the pain of family separation, one lone carer explained that she takes solace in having a family member act as her newborn’s primary carer:

*“My auntie, she can give my daughter a life I never could. She can give her anything she wants. She’ll never need for nothing, so that’s enough for me.” (Samantha)*

In this carer’s case, she maintains a healthy relationship with her auntie and this arrangement means that both of the girls are cared for within the family, albeit only one child remains in her home. Likewise, other carers, some of whom were grandparents, explained that they assumed the primary carer role to keep the children within the family.

*“Our daughter had a drug and alcohol problem and her older two children were removed by [social services], and we didn’t want them to go into foster care, so we took them. Then she seemed to get her act together for a while and had the third child, and then ended up relapsing and they removed him. And the same story with the younger one.” (Bree)*

Indeed, family support was discussed as a critical element of coping with having a child with a disability. In some cases, carers moved from distant areas to obtain more support from health services as well as to be closer to family.

In addition to providing emotional and instrumental support in the form of respite and caregiving, several family members were able to leverage their employment and social networks to facilitate access to schools, housing, and non-government organisation resources. In one example, a carer moved to a capital city to be closer to her mother and grandmother who were able to obtain support and advocacy from various organisations.

*“(My Nan) is very highly respected in the community, so she knows a lot of people. So, yeah, it’s not about what you know, it’s pretty much who you know these days*…*The [caseworker] was one of my Nan’s best friends, so she come and picked me up, picked my nan up, took me to Housing and then we sat down with the Department of Housing and they got the manager out and we told them my situation. The manager was like, ‘alright, I’ll ring you back in a week,’ and they ended up offering me a house.” (Samantha)*

In her perspective, this carer’s experiences highlighted the role of her influential, well-networked family member and caseworker friend as facilitating an unfettered path to housing. Conversely, there were several other examples of participants who did not have such resourceful networks. As previously described, some carers experienced social isolation after having moved away from family to have better service access closer to a metropolitan area or were estranged from family (Table 2). Also described were family members acting to validate concerns and difficulties or prompt to seek care. One carer explained that her mother-in-law recognized the need to seek care based on previous personal and professional experience:

*“Cause I had a lot of problems with (my daughter) beforehand, but I was kind of in denial and didn’t want people to say that she had problems. But, [my partner’s] mother had told me to come here to see a paediatrician because she knew (there was a problem).” (Rachel)*

Another woman discussed social support as the main influence on her ability to cope. Barbara described the support she received from her brother, friends, and work colleagues as integral to her coping.

*“I was lucky I had my brother. My brother’s always been with me… So I think if I didn’t have him I would have lost the plot.” (Barbara)*

These examples exemplify the influential role of the familial context in mitigating or intensifying the costs of caregiving.

## Discussion

Our data show that carers bear the costs, both economic and non-economic, of fragmented systems and complex pathways to care. Aboriginal carers’ ability to draw on family resources to care for their children with disability, despite these high costs demonstrates strength and resilience.

### Bearing the costs

In spite of the Australian context of universal health care and specific policy initiatives aimed to reduce costs of healthcare for Aboriginal and Torres Strait Islander peoples, such as Closing the Gap [25], carers in this study reported that financial costs incurred as part of their caring roles strained resources and exceeded their capacity. While financial stress previously has been reported in literature on caregiving for people with a disability [2], we have contributed the experiences and perspectives of costs and resources associated with caring for an Aboriginal and Torres Strait Islander child with a disability. Lone carers or single parents are recognised as some of the most economically disadvantaged groups in Australian society and there is a higher prevalence of lone-parenthood in Indigenous women than others [11, 33]. Our participants were all women, mostly mothers and some grandmothers, half of whom were lone carers for one or more child with a disability. Previous research has reported that single mothers with a child with a disability are less likely to be in the labour force than single mothers of a young child without a disability [34], thereby constraining income. Even small out-of-pocket expenses can contribute to financial strain in carers, particularly those whom are financially disadvantaged [35, 36]. Providers should consider the financial outlays characteristic of many care pathways and forewarn carers of service costs and caveats [22]. Concerted efforts to streamline services and systems may mitigate these costs. The experiences described by our participants depict an intersection of race, socio-economic status, gender, disability, and caregiving. Services and funding initiatives should incorporate such intersecting determinants in planning and delivery of holistic care.

In most instances, living within a context of a multiple child household challenged the lone carers’ ability to meet needs of all household members. Research on siblings of children with disability has identified siblings may witness or experience physical violence perpetrated by their sibling with a disability that makes them feel unsafe and anxious at home [37]. As in the present study, long-term alternate accommodations were sometimes sought for the non-disabled sibling to enable them educational opportunities and physical safety that were not necessarily available living at home because of the high level needs and behavioural problems of their sibling. While in some families, the child with a disability is accommodated elsewhere which enables other members of the family to have quality time spent together, in other families, the child(ren) with a disability remained at home and siblings were accommodated elsewhere.

One option for accommodation of children outside their parental home is kinship care, a formal or informal arrangement when care is provided within the family or friendship network [38]. This arrangement has been associated with greater psychological benefit [39], stability of care, more contact with parents and other family members, less trauma being separated from parents, and less stigma [40]. These are beneficial outcomes for children, but the kinship carers may not fare as well. A study of Indigenous and non-Indigenous kinship carers from urban and rural areas of New South Wales, Australia, revealed extensive and complex support needs. This was particularly the case for older carers, often grandparents, who were resuming previous caregiving roles [39]. Indigenous kinship carers were more likely to have poor health, financial hardship, and have multiple caregiving roles [39], yet were less likely to seek help from statutory agencies [41]. The Kinship Care Family Research Report concluded that needs of kinship carers included increased financial and non-financial support to caregivers of Aboriginal children, recognition of Aboriginal caregivers as more likely being older, single, have poorer health, and caring for and supporting multiple children [38]. Financial and support services may be particularly beneficial for such carers to mitigate the negative impact on health and wellbeing [42]. Despite these concerns, however, grandparent and other kinship caregivers are reported to be extremely resilient, thus demonstrating the importance of developing mechanisms to support resilience [42].

### The social capital of family

Family is a cornerstone of Aboriginal culture and includes nuclear and kinship or extended family as well as others within the community who are considered to be family, and who will potentially provide support for children throughout their lives [43]. Family functioning can influence the adverse effect of disadvantage on health and wellbeing in Aboriginal culture [44]. Good family functioning is associated with improved mental and physical health of parents/carers of a person with a disability [3] and as such, should be supported. A key finding of this study was the important role of family as a resource to enact respite, provide instrumental and emotional support, and facilitate service access and empowerment of carers. Use of social and familial networks to obtain resources and facilitate access is a universal behaviour, yet it should be appreciated that some carers may not be networked and thus are vulnerable to isolation and obstacles to needed support.

Our findings indicate that respite is perceived as a family matter. Participants reported that they did not trust ‘outsiders’ with providing the same level of care that the family can provide. It is therefore particularly important that family kinship systems be supported in their roles as respite providers for carers [1]. For carers who are un-networked and vulnerable, provision of support mechanisms should be specifically targeted to their needs to facilitate resilience [45].

### Facilitating networks

Formal and informal social support is beneficial to being able to cope as well as mitigate stress and some of the negative health impacts from caring for a child with a disability. Several participants voiced their desire for opportunities to speak, share experiences, and garner strength from linking with other Aboriginal family carers of children with a disability. Participants in support groups can gain emotional and practical information, experiential knowledge, and empathy via reciprocity of peer support [46]. Such groups can provide a sense of community, unconditional acceptance and information, and help to facilitate relationships with family and friends [4]. There is a dearth of information on how Aboriginal carers of children with a disability can best be supported through their disability journey by culturally-appropriate groups and whether this might be one way of overcoming the challenges and disparities faced by Aboriginal Australians.

### Limitations

The small self-selected sample preclude generalisation to other Aboriginal and Torres Strait Islander populations or to indigenous populations more widely. Yet, these information-rich narratives were elicited from participants who were purposively recruited from a developmental clinic sample, and as such, the findings may be transferable to individuals in that type of setting. Clinic staff enacted a type of gatekeeping role during recruitment by alerting the researcher to eligible participants who were attending the fortnightly clinic. Being recruited from this clinic may mean that participants were more likely to be receiving care for their child and their access challenges were different because of the increased support or other circumstances related to being a patient of the health service. For example, some participants used the transport service provided by the facility to attend the clinic appointment. Interviews were conducted at an Aboriginal health service, by individuals with organisational affiliations which may have inhibited disclosure of information regarding experiences with that service. Only women carers’ perspectives were represented, so future research should seek out male carer perspectives.

## Conclusions

Addressing the needs of carers of Aboriginal and Torres Strait Islander children with a disability is vital in the shift to providing community-based care for people and families living with disability. Tailoring services to these carers’ needs means supporting kinship caregiving, engaging with other Aboriginal families, and the needs of Aboriginal women who are lone carers and especially vulnerable to financial strain and social isolation. Family resources can mitigate the costs associated with caring for a child with a disability in Aboriginal families and facilitate resilience.

## Abbreviations

AH&MRC: Aboriginal Health and Medical Research Council
GP: General practitioner
OOHC: Out-of-home care
